# Ecofriendly Mortar with Paint Sludge Ash

**DOI:** 10.3390/ma18092080

**Published:** 2025-05-01

**Authors:** Solomon Asrat Endale, Mitiku Damtie Yehualaw, Woubishet Zewdu Taffese, Duy-Hai Vo

**Affiliations:** 1 Faculty of Civil and Water Resource Engineering, Bahir Dar Institute of Technology, Bahir Dar University, Bahir Dar P.O. Box 26, Ethiopia; bdu1305626@bdu.edu.et; 2 Department of Civil, Architectural and Environmental Engineering, Missouri University of Science and Technology, Rolla, MO 65401, USA; 3 School of Research and Graduate Studies, Arcada University of Applied Sciences, Jan-Magnus Jansson Aukio 1, 00560 Helsinki, Finland; 4 Department of Materials Science and Engineering, Missouri University of Science and Technology, Rolla, MO 65401, USA; dvnm6@mst.edu

**Keywords:** cement partial replacement, paint sludge ash, material characterizations, fresh properties, mechanical properties, durability performance, microstructural analysis, ecofriendly mortar

## Abstract

This research aims to address the environmental and economic challenges associated with conventional concrete by partially replacing cement—the most polluting, expensive, and energy-intensive ingredient—with industrial paint sludge ash (PSA), a highly contaminated industrial waste that is typically landfilled. Mortar mixtures were prepared with PSA replacement levels ranging from 0% to 20% in 5% increments while maintaining a constant water-to-binder ratio of 0.48. This study comprehensively evaluated the fresh, mechanical, durability, and microstructural properties of the PSA-modified mortar to assess its potential as an ecofriendly construction material. Results showed that as PSA content increased, the fresh properties, such as workability/slump flow and setting time, decreased, while the water demand for attaining normal consistency increased. Soundness tests indicated expansion up to 15% PSA replacement, beyond which expansion became more pronounced. Compressive strength improved significantly with PSA replacements of 5% to 15% compared to the control sample, with a slight decline at 15% relative to 5% and 10%. This trend was consistent with bulk density and ultrasonic pulse velocity measurements. Furthermore, the incorporation of PSA enhanced key durability properties, including water absorption, sulfate resistance, and porosity reduction, up to 15% PSA replacement. Microstructural analysis using SEM, XRD, TGA/DTA, and FTIR confirmed that PSA inclusion led to increased mortar densification, with the 10% PSA mix exhibiting thermal stability and minimal mass loss at elevated temperatures. FTIR spectra further indicated improved composition with higher PSA content. Overall, PSA proved to be a viable partial cement replacement, offering enhanced mortar properties without compromising performance. Its use contributes to sustainability by reducing reliance on cement, lowering construction costs, and eliminating the environmental and logistical burdens of paint sludge disposal.

## 1. Introduction

Cement is one of the most widely consumed materials globally, with annual consumption exceeding 4.13 Gt and projected to reach 4.68 Gt/year by 2050 [1]. However, the environmental footprint of cement production is substantial, contributing approximately 750 kg of CO_2_ per ton of cementitious products produced. Cement manufacturing is responsible for nearly 8% of global CO_2_ emissions, which is equivalent to 5% of total greenhouse gas emissions [1,2]. Recognizing the urgency of mitigating climate change, the United Nations (UN) has introduced the Sustainable Development Goals (SDGs), including SDG 11, which promotes sustainable cities, and SDG 13, which advocates for climate action by regulating CO_2_ emissions [3,4]. Additionally, the Kyoto Protocol, which has been signed by most nations, further emphasizes the need for reducing CO_2_ emissions [4]. These global commitments highlight the necessity of developing alternative and sustainable construction materials that reduce environmental impact while ensuring performance and cost-effectiveness.

Portland cement, despite its widespread use, remains an energy-intensive material that significantly impacts project costs, particularly in developing countries where its price is high due to large-scale usage [5]. To address these issues, the construction industry has shifted its focus toward innovative materials that serve as full or partial replacements for cement [6,7]. One of the most promising strategies involves utilizing industrial by-products and waste materials as supplementary cementitious materials (SCMs), which contribute to sustainable and ecofriendly concrete and mortar production.

Simultaneously, rapid industrialization has led to severe environmental challenges, particularly in developing nations, where industries discharge waste into rivers and landfills, thereby exacerbating pollution [8,9,10,11,12]. With growing concern about reducing environmental pollution, strict regulations have been imposed worldwide to ensure the proper disposal of solid and liquid waste from industries and to reduce CO_2_ emissions in the atmosphere [3]. The application of industrial sludge in agriculture faces increasing challenges due to difficulties in standardizing quality and concerns about heavy metal contamination [13]. While researchers have attempted to repurpose sludge in construction materials [10,11,12,14,15,16], there remains a need for more extensive investigations into its potential as a viable SCM. 

Among industrial wastes, paint sludge represents a particularly underexplored and problematic stream. The automotive industry, for instance, generates 2.5–5 kg of paint sludge per painted vehicle—translating to 2500–5000 tonnes annually for a mid-sized manufacturer producing 100,000 vehicles [17]. This hazardous by-product, consisting of paint particles, solvents, binders, pigments, and additives, poses serious health and environmental threats when inadequately disposed of through landfilling or incineration [13,18,19,20,21]. While there has been increasing adoption of waste materials from construction, agriculture, and other industrial processes [22,23,24,25,26,27], the reuse of paint sludge in the cement sector remains largely unexplored.

A promising and more sustainable alternative lies in incorporating processed paint sludge into cementitious systems. When treated and converted into ash—typically via incineration—paint sludge can enhance binding properties and potentially serve as a partial cement replacement. Studies have shown that incorporating powder derived from automotive or waste latex paint sludge can improve compressive strength and reduce porosity and water absorption, up to an optimal replacement level [10,15,18,19,20,28,29,30,31,32].

Despite its potential, limited research has been conducted on the direct incorporation of raw paint sludge powder (without calcination) into concrete, cement-based materials, admixtures, clay bricks, or other applications [17,18,19,20,29,30,33,34,35]. Thus, existing studies have primarily focused on unprocessed paint sludge, which limits its reactivity. Most existing studies have focused on untreated sludge, which exhibits limited pozzolanic reactivity. Calcination at controlled temperatures is crucial for transforming paint sludge into a reactive amorphous ash that meets ASTM C618 [36] criteria for pozzolans—specifically, a minimum combined SiO_2_, Al_2_O_3_, and Fe_2_O_3_ content of 70% [36,37,38]. Raw sludge typically falls short, with siliceous contents ranging between 30% and 40% [32,39,40], further justifying thermal treatment.

Although calcination increases energy use, comparative assessments suggest that the overall environmental impact of producing paint sludge ash (PSA) is lower than that of conventional cement clinker production [1,41]. Given that paint sludge is among the most environmentally burdensome and difficult-to-manage industrial wastes, transforming it into a value-added material not only reduces carbon emissions but also alleviates landfill dependency, minimizes land acquisition costs, and provides a sustainable solution for solid waste management in the paint manufacturing industry.

Nevertheless, the existing body of literature lacks comprehensive and systematic investigations into the long-term performance of PSA in cement-based materials, especially with respect to mechanical strength, durability, and microstructural characteristics. In particular, the influence of varying PSA replacement levels and curing durations on these properties remains insufficiently understood.

This study aims to address these knowledge gaps by evaluating the feasibility of utilizing PSA as a partial replacement for cement in mortar production. The primary objective is to develop ecofriendly, cost-effective mortar that reduces CO_2_ emissions and offers an innovative solution for industrial paint sludge disposal. The experimental program involves mortar mixtures incorporating 5%, 10%, 15%, and 20% PSA, with curing durations spanning from 3 to 91 days. A comprehensive suite of tests was conducted to assess fresh properties, mechanical strength, durability performance, and microstructural characteristics. By advancing the understanding of PSA’s role in mortar production, this study contributes to the broader goals of sustainable construction, environmental conservation, and the circular economy. It establishes a foundation for future research and potential large-scale applications of industrial waste-derived materials in civil infrastructure.

## 2. Materials and Methods

This section outlines the materials utilized, the mix design, and the testing methodologies employed in this study.

### 2.1. Materials

#### 2.1.1. Cement

The cement used for all mortar mixes was Ordinary Portland Cement (OPC) with a grade of 42.5 N. The physical properties of the cement were verified to confirm compliance with ASTM standards. All measured parameters were within permissible limits, ensuring their suitability for mortar production.

#### 2.1.2. Paint Sludge Ash

The paint sludge used in this study was collected from an effluent treatment plant, specifically from a sludge drying bed, which is a by-product of the production of water-based and quartz paints. To ensure a representative sample, sludge was collected from different depths (upper, middle, and lower layers) of the drying bed. Prior to testing, the sludge was thoroughly mixed to achieve homogeneity and sun-dried to remove excess moisture. The dried sludge was then subjected to chemical and physical characterization before being incorporated into the mortar mixes.

The production method used in this study was developed based on a review of the relevant literature and trial tests analyzing the chemical composition of PSA. In previous research, raw automotive or latex paint sludge powder has been used in concrete and other applications without incineration, relying solely on oven drying of the sludge [11,18,32,42,43,44,45]. However, this approach limits the material’s reactivity [37,38]. Thus, the findings of the trial tests conducted in this study served as the basis for determining the optimum burning temperature for PSA production. Although incinerating paint sludge at elevated temperatures contributes to CO_2_ emissions, this impact is mitigated when compared to the energy and emissions associated with conventional cement clinker production. Additionally, using PSA offers a sustainable solution for managing hazardous paint sludge waste, thereby reducing environmental pollution and supporting circular economy principles.

The initial processing involved sun-drying the paint sludge, followed by oven drying at 105 °C for 24 h. The dried sludge was then incinerated in a controlled combustion furnace at three different burning temperatures (700 °C, 750 °C, and 800 °C for 2 h) to evaluate its reactivity and structural properties. Based on the trial results, 750 °C was identified as the optimal burning temperature, producing PSA with a high amorphous content, a high percentage of siliceous material, and maximum compressive strength at early curing ages (3 and 7 days). This outcome aligns with ASTM C618 [36], which specifies requirements for pozzolanic materials.

**Figure 1:**
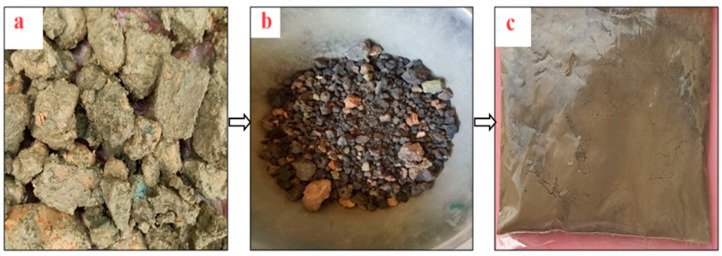
Stages of PSA production: (**a**) raw paint sludge, (**b**) oven-dried and calcined sludge, and (**c**) ground PSA powder.

The final bulk production method involved collecting sludge from the effluent treatment plant, allowing it to air-dry, then oven drying at 105 °C for 24 h, followed by calcination at 750 °C for 2 h with a heating rate of 10 °C per minute and natural cooling. The calcined material was then ground in a ball mill at 70 rpm for 1 h to achieve the desired particle gradation. Figure 1 presents the production stages of PSA.

**Figure 2:**
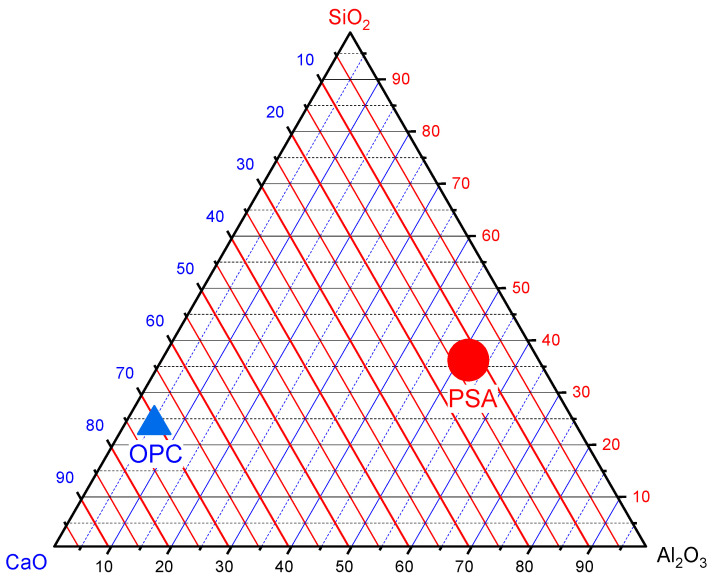
Ternary diagram of OPC and PSA.

The chemical composition of PSA was analyzed using X-ray fluorescence (XRF) in accordance with ASTM C618 and ASTM C150 [36,46]. The results show that PSA is a low-calcium material (CaO = 5.98%) with a high siliceous content (72.33%), comprising SiO_2_ (17.65%), Al_2_O_3_ (25.19%), and Fe_2_O_3_ (29.49%). According to ASTM C618 [36], materials with SiO_2_ + Al_2_O_3_ + Fe_2_O_3_ ≥ 70% are classified as Class N pozzolans, confirming PSA’s suitability as a supplementary cementitious material (SCM). The loss on ignition (LOI) of 5.28% is well below the ASTM limit of 10%, indicating efficient combustion and minimal unburned carbon. Table 1 and Figure 2 present the oxide composition and ternary diagram of OPC and PSA.

A summary of the physical properties of OPC and PSA is presented in Table 2. The bulk density of the PSA was measured at 2157 kg/m^3^, which is significantly higher than that of OPC (1440 kg/m^3^). This finding contradicts previous studies [29,30,32], in which the bulk density of raw, uncalcined paint sludge was reported to be lower than that of OPC. This discrepancy can be attributed to differences in production methods, calcination temperatures, grinding times, and sludge sources, which affect the final density of the material.

The specific gravity of PSA was found to be 2.76 g/cm^3^, which is lower than that of OPC (3.15 g/cm^3^). This trend aligns with findings from previous studies [12,15,32], confirming that PSA has a lower specific gravity than OPC, even in its raw form. The Brunauer–Emmett–Teller (BET) surface area analysis revealed that PSA has a higher specific surface area (490 m^2^/g) compared to OPC (340 m^2^/g). A larger surface area enhances chemical interactions with water, accelerating the hydration process and improving strength development [37,38].

**Figure 3:**
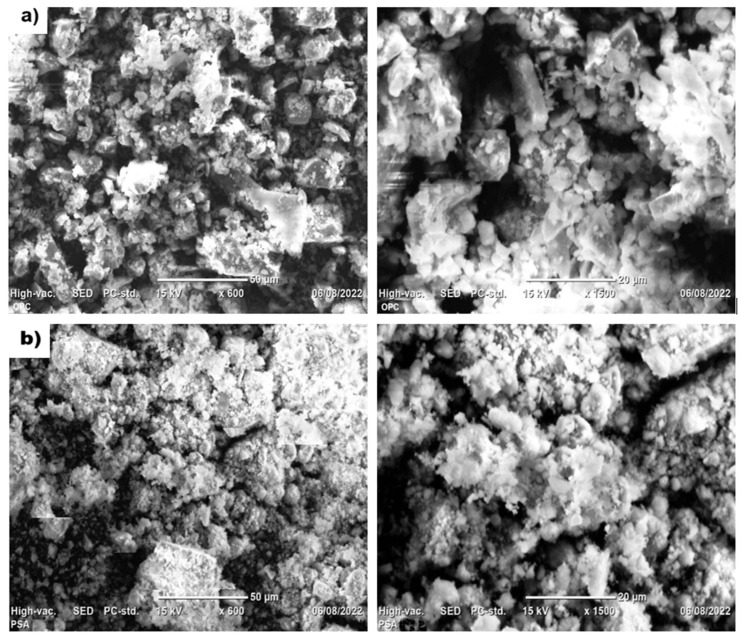
SEM image of (**a**) OPC and (**b**) PSA with 600×, and 1500× resolutions.

SEM was conducted under high vacuum at 15 kV, revealing the surface morphology of PSA powder. As shown in Figure 3, the PSA particles exhibit an irregular and porous structure with angular formations. The microstructure of automotive or other paint sludge powder depends on the type of paint produced and the processing methods used for its treatment, as well as the production methods of the ash [9,15,42,44,47]. For comparison, the OPC powder used in the mortar mix also exhibited an irregular morphology but had a higher volume of internal voids and similarly angular particle shapes. These morphological characteristics may influence the packing density, reactivity, and interfacial behavior in cementitious systems.

Previous studies [11,19,28,32,44] have reported similar findings, confirming that paint sludge powder has a porous and irregular surface, with variations in particle size and diameter. However, due to calcination and grinding strategies, the PSA in this study exhibited a denser and more reactive surface, which is suitable for pozzolanic reactions. 

**Figure 4:**
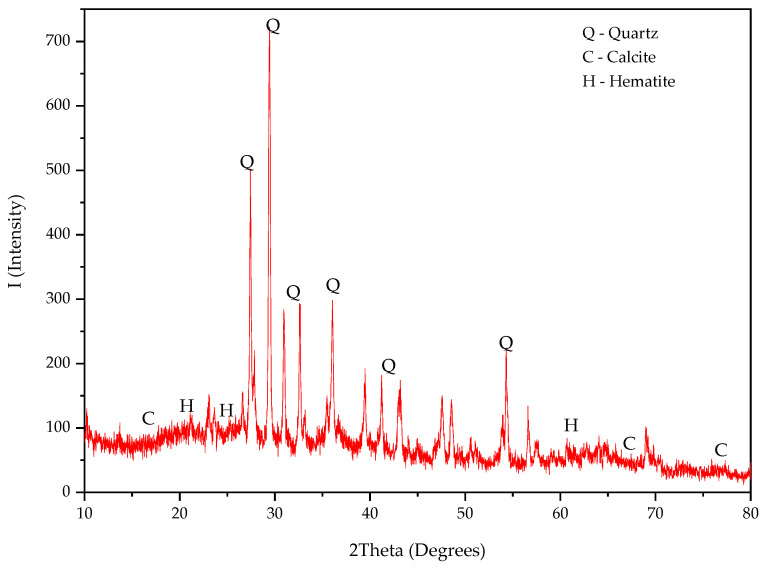
XRD pattern of PSA.

XRD analysis was performed using the random powder method at 2θ of 10–80^o^ to determine the mineral phases present in PSA. As shown in Figure 4, PSA exhibited both crystalline and amorphous phases, with quartz (SiO_2_) as the primary crystalline phase. The presence of calcite (CaCO_3_) and hematite (Fe_2_O_3_) was also observed, indicating the formation of amorphous calcium- and iron-based phases.

Comparatively, other studies using raw paint sludge (without calcination) have identified minerals such as titanium oxide, alite, belite, calcite, ettringite, and portlandite [28,31,34,48]. However, the high-temperature incineration process used in this study led to a more reactive amorphous phase, enhancing PSA’s potential as a cement replacement material.

#### 2.1.3. Fine Aggregate

**Figure 5:**
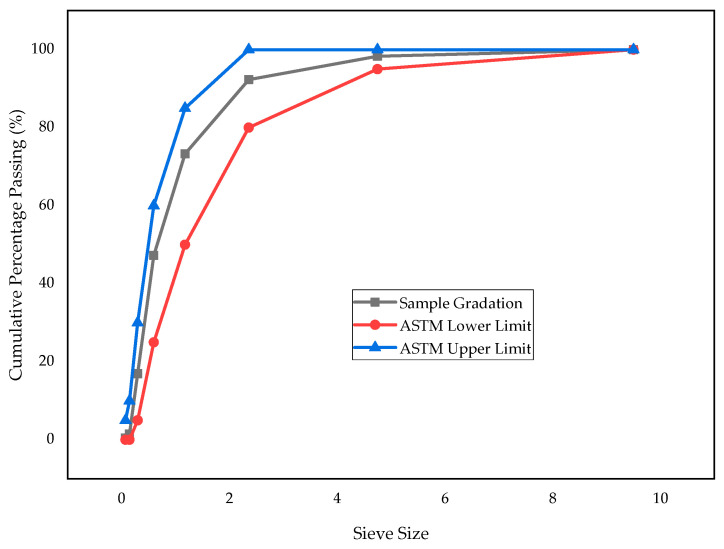
Gradation curve of sand.

Locally available natural river sand was used as the fine aggregate. The sand was well-graded, washed, and free from dust and deleterious materials to ensure consistency in mortar production. A comprehensive assessment of the sand’s physical properties was conducted, and the results are summarized in Table 3. The sand’s grading curve, illustrated in Figure 5, was found to be within the upper and lower limits specified by ASTM C136 standards [49].

#### 2.1.4. Mixing Water

For mortar preparation, potable tap water that was colorless, odorless, and free from impurities was used. This ensured that no external contaminants influenced the hydration process of the cementitious materials.

### 2.2. Experimental Methods

#### 2.2.1. Mix Proportions and Sample Preparation

The collected paint sludge and processed ash samples were systematically organized, labeled, and stored under dry conditions. Given the significant material requirements for concrete production, this study was conducted using mortar mix samples instead. The ACI method was employed to determine the proportions of the mortar ingredients, including OPC, PSA, sand, and water. 

Mortar mixing uses a weight ratio of 1 part cement to 2.75 parts sand to prepare mortar cubes measuring 50 mm × 50 mm × 50 mm. The mixing procedure adhered to ASTM C305 guidelines [54]. A series of mortar mixes were prepared with PSA replacement levels ranging from 0% to 20% (in 5% increments) while maintaining a constant water-to-binder ratio of 0.48, as determined through trial tests. Table 4 presents the mix proportions used in this study.

Trial compressive strength tests indicated that PSA replacement of up to 10% resulted in improved compressive strength compared to the control sample. Therefore, mixes with PSA levels of up to 20% were prepared to examine performance trends across different curing periods. Curing was conducted in accordance with ASTM C192 [55] guidelines. A total of 315 mortar cubes were cast and distributed as follows: 105 mortar cubes for compressive strength, ultrasonic pulse velocity (UPV), and microstructural analysis; 105 cubes for water absorption, dry density, and porosity/voids; and 105 cubes for sulfate attack resistance or compressive strength loss testing. All tests were conducted for PSA replacement levels of 0%, 5%, 10%, 15%, and 20% at curing ages of 3, 7, 14, 21, 28, 56, and 91 days.

#### 2.2.2. Test Methods

The engineering properties of PSA were evaluated before production to determine their influence on mix performance and quality. The material characterizations of PSA and OPC powder were assessed through various physical, chemical, microstructural, and mineralogical analyses, including X-ray fluorescence (XRF), scanning electron microscopy (SEM), and X-ray diffraction (XRD). These tests were conducted to determine the chemical composition, microstructure, and mineralogical characteristics of the materials, ensuring compliance with ASTM standard specifications. Table 5 presents a summary of the test methods and standards used in this study.

The fresh properties of the mortar were evaluated using ASTM standard tests, including workability/slump flow (ASTM C1437 [55]), consistency (ASTM C187 [57]), setting time (ASTM C191 [58]), and soundness (ASTM C151 [59]). These tests ensured that the fresh-state behavior of PSA-modified mortar met the performance criteria for construction applications.

In terms of mechanical properties, mortar cube specimens were tested for compressive strength, UPV, and bulk density at different curing ages, following ASTM standards. Compressive strength was assessed according to ASTM C109 [60], while UPV measurements were conducted according to ASTM C597 [61] to evaluate the quality and internal defects of the mortar. Bulk density was determined using ASTM C138 [62], with specimens weighed in a saturated surface dry (SSD) condition.

Durability tests were conducted to assess long-term performance, including water absorption, sulfate attack resistance, and porosity/voids analysis, in accordance with ASTM standards. Water absorption was measured according to ASTM C642 [63], where dried mortar samples were immersed in water, and mass gain was calculated as a percentage of the dry mass. Sulfate resistance was evaluated by immersing specimens in a 5% sodium sulfate (Na_2_SO_4_) solution and monitoring compressive strength loss over time according to ASTM C1012 [64] and methodologies reported in related studies [24,65]. Compressive strength loss was monitored over time, and sulfate resistance was quantified by comparing the compressive strength of specimens cured in a sulfate solution to those cured in potable water.

Porosity, or void volume, was determined by calculating the ratio of pore volume to total specimen volume. This involved measuring the mass of the specimens in air and when submerged in water, which provided an estimate of the internal voids affecting permeability and durability.

The microstructural evolution of the mortar samples was analyzed at 7 and 28 days of curing using SEM, XRD, Thermogravimetric Analysis and Differential Thermal Analysis (TGA/DTA), and Fourier-Transform Infrared Spectroscopy (FTIR). SEM provided insights into morphological changes and hydration products, while XRD was used to identify mineralogical phases and crystalline structures. TGA/DTA assessed mass loss and thermal stability across a temperature range of 20 °C to 900 °C, increasing at a rate of 20 °C per minute using alumina crucibles. FTIR analysis was conducted using a JASCO 6660 FTIR spectrometer (Tokyo, Japan) in transmittance mode, covering a frequency range of 4000 cm^−1^ to 400 cm^−1^, to identify functional groups and chemical bonding.

## 3. Results and Discussion

### 3.1. Effects of PSA on the Fresh Properties of Mortar

#### 3.1.1. Slump Flow 

**Figure 6:**
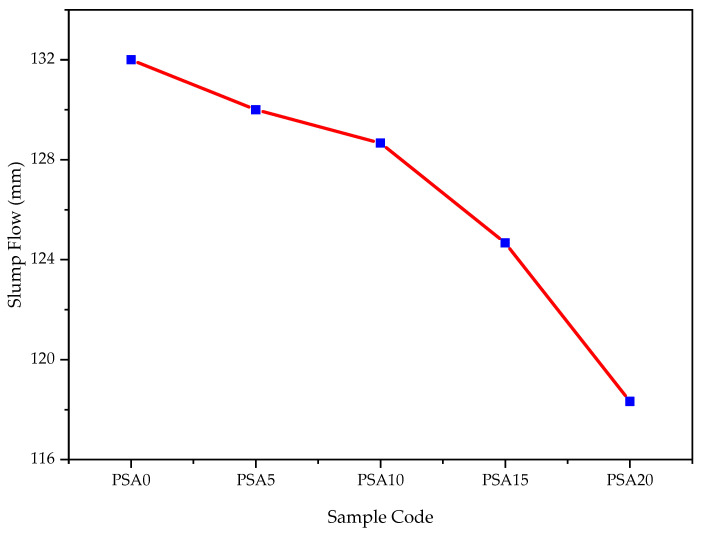
Slump flow of mortar results with different percentages of PSA.

The slump flow of mortar was observed to decrease with increasing PSA content, as shown in Figure 6. The control mortar exhibited the highest slump flow of 132 mm, which is attributed to its lower water demand and the surface area of OPC particles. In contrast, mortar mixes incorporating 5%, 10%, 15%, and 20% of PSA showed reductions of 1.51%, 2.52%, 5.88%, and 10.35%, respectively. Despite these reductions, all slump flow values remained within the acceptable range specified by ASTM C1437 [56], which requires a flow rate of 110 ± 5% for satisfactory mortar workability.

The decrease in slump flow is primarily due to the porous nature of PSA, which results in a larger specific surface area that requires more water for proper lubrication and flow, as observed in related materials [37,38,66]. Moreover, the irregular and angular morphology of PSA particles, as confirmed by microstructural analysis, increases internal friction within the mortar mix, further reducing workability. This observation aligns with previous research that highlights the significant influence of particle size and surface texture on the fresh properties of cementitious materials [23,67]. The absorption of mixing water by PSA particles reduces the amount of free water available, leading to a lower slump compared to the control mix. This suggests that to maintain the same consistency as conventional mortar, chemical admixtures (other than water-reducing agents) may be required. Similar effects have been reported for mortars incorporating automotive paint sludge and waste latex paints as cementitious components [9,29,30,33]. Interestingly, Almesfer and Ingham [28] reported that waste latex paint, when used as a substitute for mixing water or as a chemical additive, could improve cement paste workability. This indicates that the complex interactions between PSA and other mix constituents warrant further investigation to optimize fresh performance.

#### 3.1.2. Consistency and Setting Time

**Figure 7:**
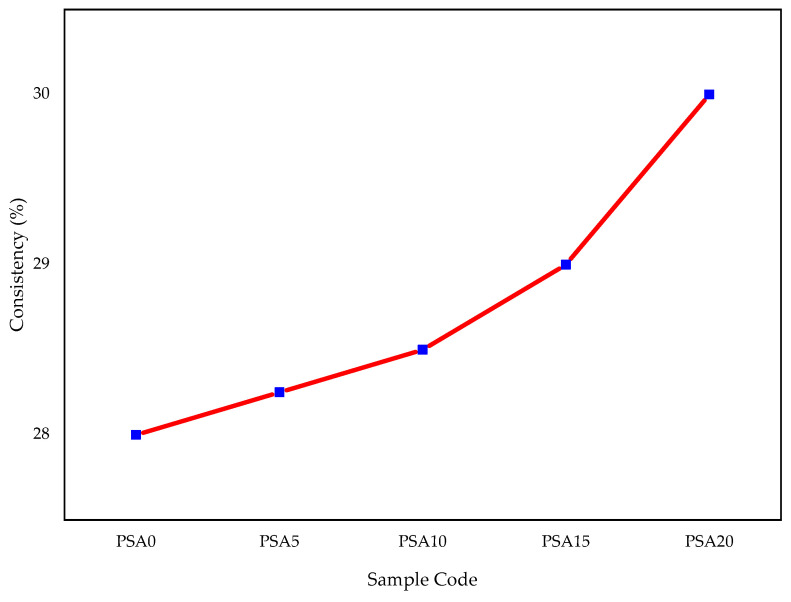
Cement consistency results with different percentages of PSA.

The normal consistency of cement paste increased with higher PSA replacement levels, as illustrated in Figure 7. The 5%, 10%, 15%, and 20% PSA replacements resulted in increases of 1.78%, 3.57%, and 7.14%, respectively. Despite these increases, all values remained within the acceptable limits of ASTM C187 (26–33%) [57]. This increased water demand can be attributed to the higher surface area and porosity of PSA particles, which absorb more water than OPC. The BET surface area analysis confirmed that PSA exhibits a larger surface area compared to OPC, which directly influences its water demand.

Consistent trends have been observed in prior studies, where increasing the amount of automotive and waste latex paint sludge powder led to higher water requirements due to its greater surface area, lower specific gravity, and increased surface roughness [9,28,32]. Additionally, due to the particle size of PSA, greater resistance was observed when using the Vicat apparatus to determine consistency, requiring additional water to achieve normal consistency. A lower specific gravity may also contribute to an increased paste volume, necessitating more water to maintain workability [32].

**Figure 8:**
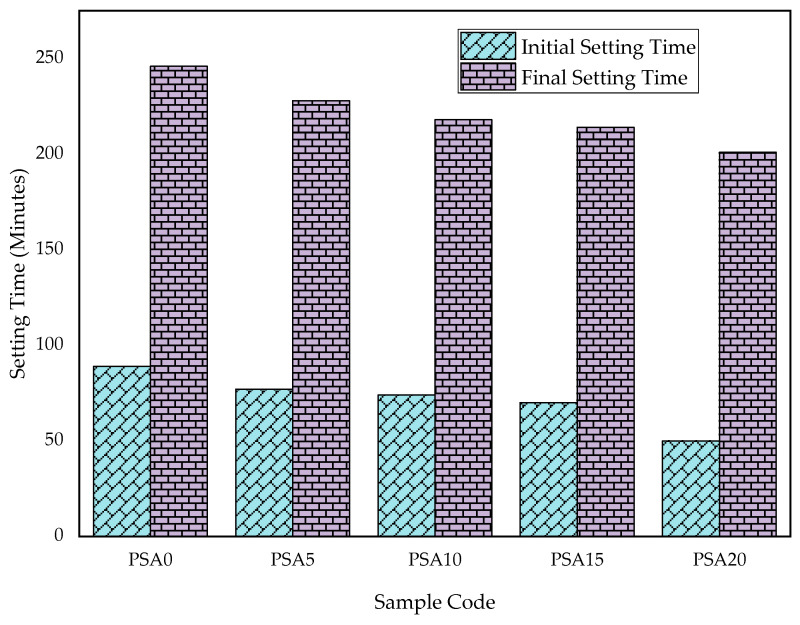
Cement setting time results with different percentages of PSA.

The setting time of PSA-incorporated mortar was also evaluated, as shown in Figure 8. Both the initial and final setting times decreased with increasing PSA replacement, ranging from 50 to 89 min for the initial setting time and from 201 to 246 min for the final setting time. These values comply with ASTM C191 standards, which specify an acceptable range of 49–202 min for initial setting and 185–600 min for final setting [58].

Similar findings have been reported, where replacing OPC with waste latex paint sludge powder led to reduced setting times [28,33]. However, some studies have reported contrary results, indicating that both the initial and final setting times are increased with higher paint sludge content [32]. This was attributed to a reduction in tricalcium silicate (C_3_S) content, which is primarily responsible for early strength gain in OPC. The use of automotive paint sludge slows down the hydration process, delaying the setting process due to the reduced C_3_S content [42,68]. The slower pozzolanic reaction of PSA, compared to OPC components, further supports this explanation.

#### 3.1.3. Soundness

**Figure 9:**
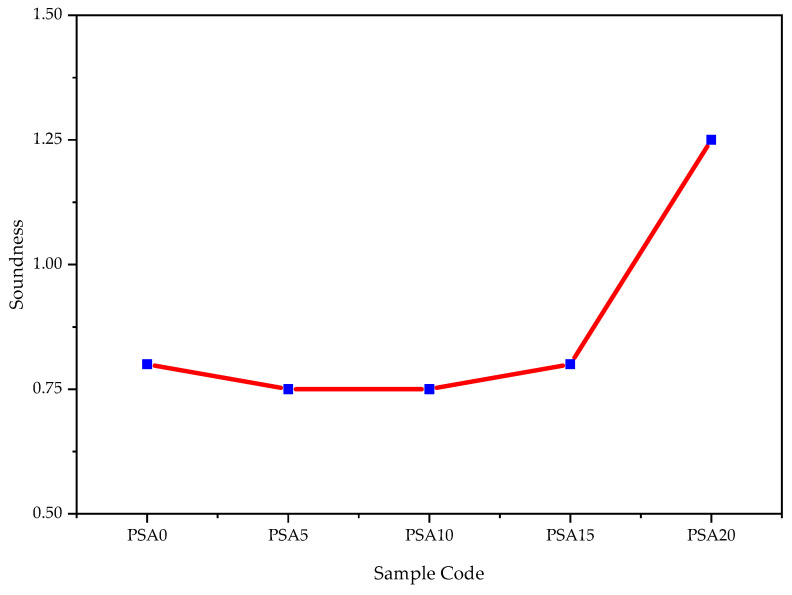
Cement soundness results with different percentages of PSA.

The soundness of cement refers to its ability to maintain volume stability during setting and hardening. As shown in Figure 9, the soundness of mortar incorporating PSA was lowest at 5% and 10% replacement levels, while at 15% PSA replacement, it exhibited a similar increase in expansion, indicating greater instability at higher replacement levels. 

The initial reduction in expansion at lower PSA contents may be attributed to the presence of minor quantities of calcium oxide (free lime, f-CaO) and magnesium oxide (periclase, MgO), which, when present in controlled amounts, can reduce deleterious expansion effects [65,69]. This trend is consistent with findings from previous studies, which reported that increasing the percentage of paint sludge powder can result in reduced volume expansion. Notably, the incorporation of water-based raw paint sludge powder was shown to enhance soundness by minimizing the potential for crack formation due to volumetric changes [19,32].

### 3.2. Effects of PSA on the Mechanical Properties of Mortar

#### 3.2.1. Compressive Strength

**Figure 10:**
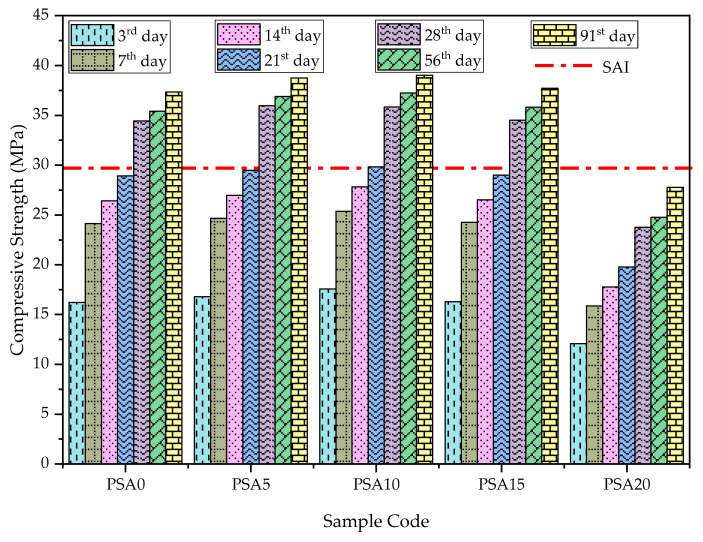
Compressive strength results of PSA-containing mortars.

The effect of PSA as a partial cement replacement on compressive strength at different curing ages is presented in Figure 10. The results indicate that mortar mixes containing 5%, 10%, and 15% PSA exhibited higher compressive strength than the control mix at all curing ages. However, beyond a 15% replacement, compressive strength decreased significantly. Although the 15% PSA replacement showed an increase in compressive strength compared to the control sample, it was lower than the strengths observed for the 5% and 10% PSA mixes.

As depicted in Figure 10, the compressive strength increased with curing time, with the 10% PSA mix exhibiting the highest strength across all levels, achieving 35.83 MPa on the 28th day and 39.02 MPa on the 91st day. In contrast, the 20% PSA replacement exhibited the lowest compressive strength, recording 23.77 MPa and 27.77 MPa on the 28th and 91st days, respectively. Compared to the control sample, compressive strength increased by 4.45%, 4.07%, and 0.21% at 5%, 10%, and 15% PSA replacement levels on the 28th day, while on the 91st day, the increases were 3.79%, 4.51%, and 1.07%, respectively. The 20% PSA mix retained 69.02% and 74.38% of the control’s strength on the 28th and 91st days, respectively.

The increase in compressive strength at optimal PSA replacement levels aligns with previous studies, which reported that incorporating automotive paint and waste latex paints in cement-based materials enhances strength development up to an optimum level [14,15,32,43]. However, exceeding this limit leads to a substantial decline in strength, necessitating the careful selection of replacement ratios. The strength improvement can be attributed to the formation of secondary calcium silicate hydrate (C-S-H) gel, resulting from the pozzolanic reaction of PSA with calcium hydroxide from cement hydration. This gel refines the pore structure, leading to a denser internal microstructure, which ultimately enhances compressive strength, UPV, and durability properties [24,37,38].

Interestingly, the incinerated PSA used in this study outperformed uncalcined forms of paint sludge—such as automotive, latex, or other paint wastes—used in earlier research, whether as a partial cement replacement or as an admixture [12,19,28,30,32]. This suggests that incineration enhances the reactivity and overall contribution of PSA to strength development.

To assess the pozzolanic properties of PSA, the Strength Activity Index (SAI) was evaluated according to ASTM C618, which defines SAI as the ratio of the compressive strength of pozzolan-containing mortar to that of the control at a specific curing age [36]. The SAI results for PSA-incorporated mortars are presented in Table 6. The 5%, 10%, and 15% PSA mixes achieved SAI values ≥1.00 at all curing ages, indicating superior performance compared to the control. However, the 20% PSA replacement exhibited lower SAI values (ranging from 0.66 to 0.74), suggesting it retained at least 66% and 74% of the control’s strength at 7 and 28 days of curing, respectively.

According to ASTM C618, all classes of pozzolan replacement must achieve SAI ≥ 0.75 on both the 7th and 28th days for satisfactory performance [36]. The findings indicate that mixes with up to 15% PSA replacement meet this criterion. The higher SAI values at later curing ages confirm the pozzolanic nature of PSA, which reacts with Ca(OH)_2_ to form additional C-S-H gel, contributing to continued strength development over time.

#### 3.2.2. Ultrasonic Pulse Velocity (UPV)

**Figure 11:**
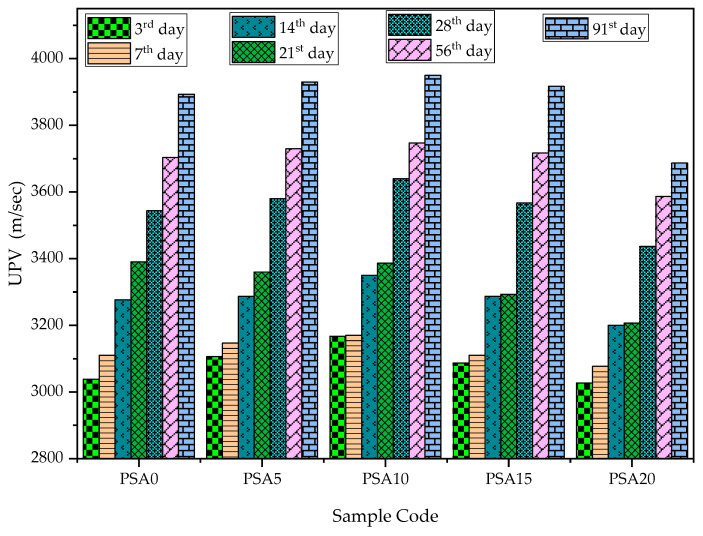
UPV results of PSA-incorporated mortars.

The UPV for PSA-incorporated mortars is shown in Figure 11. The results indicate that UPV values increased with PSA content up to 15%, with the 5% and 10% PSA replacements exhibiting the highest UPV values. However, at 15% PSA, the UPV began to decrease relative to the 5% and 10% replacements. The UPV ranged from 3.03 km/s to 3.95 km/s (3026.67 m/s to 3950.00 m/s), increasing as the curing age progressed. 

The UPV trends closely aligned with compressive strength and bulk density values, reinforcing the positive effect of PSA incorporation at optimal levels. The increase in UPV can be attributed to the formation of additional C-S-H gels, which enhance mortar density and reduce internal defects, ultimately improving mechanical properties [24,37]. However, at higher PSA replacements (>15%), excessive substitution led to microstructural disruptions, reducing UPV across all curing ages.

To evaluate concrete quality based on UPV, BS 1881: Part 203 [70], which classifies concrete based on uniformity and density, was used. According to this standard, UPV values of 3–3.5 km/s (after 28 days) indicate ‘Medium’ quality, and UPV values of 3.5–4.5 km/s (after 91 days) indicate ‘Good’ quality. The results confirm that PSA-modified mortar met the required quality standards, with improvements observed at longer curing ages.

#### 3.2.3. Bulk Density

**Figure 12:**
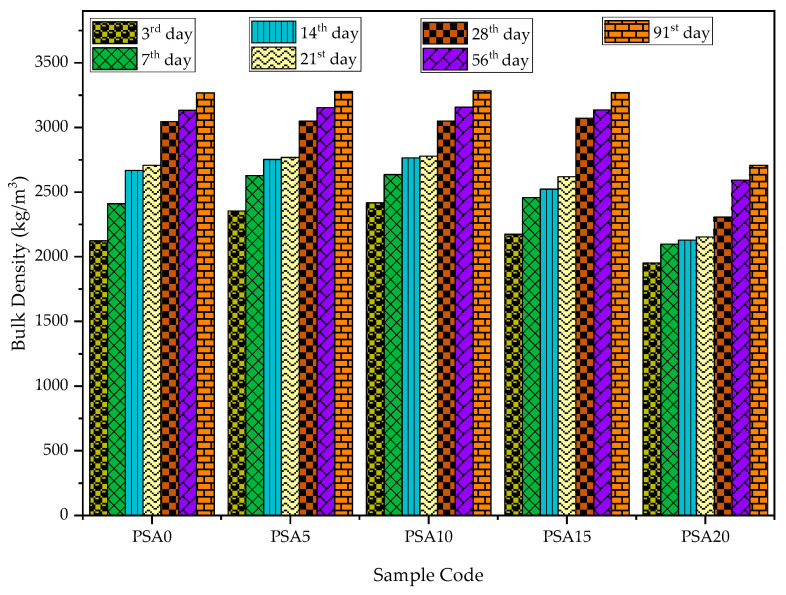
Bulk density results of PSA-containing mortars.

The bulk density of PSA-incorporated mortar at various curing ages is presented in Figure 12. The results indicate that bulk density increased with curing age and PSA replacement up to 10%, where it reached its peak performance. At 15% PSA, bulk density remained higher than that of the control but lower than the mixes with 5% and 10% PSA. The highest bulk density is attributed to the larger specific surface area of PSA, which enhances packing density and reduces porosity over time. 

Compared to the control, 5%, 10%, and 15% PSA mixes exhibited higher bulk density at all curing ages. Notably, the 10% PSA mix achieved the highest density on the 91st day (3283.24 kg/m^3^), surpassing the control. Conversely, the 20% PSA mix exhibited the lowest density (1951.29 kg/m^3^ on the 3rd day), reducing to 75.71% of the control’s density on the 28th day and 82.84% on the 91st day. These findings align with studies suggesting that automotive or other waste paint sludge content used as cement-based materials, with higher sintering temperatures or as an admixture, contributes to density improvement [30,44], while the excessive addition of waste paint sludge contributed to the reduction in density.

**Figure 13:**
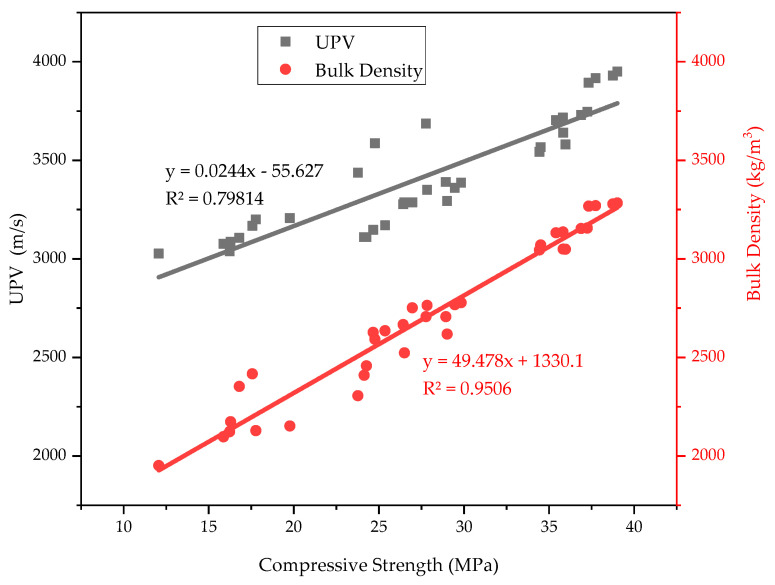
Correlation among compressive strength, UPV, and bulk density of PSA-containing mortars.

Overall, from the mechanical performance results, a strong correlation was observed among compressive strength, UPV, and bulk density, as shown in Figure 13. Statistical analysis revealed a direct and positive correlation between these parameters, with R^2^ values of 0.79814 (compressive strength vs. UPV) and 0.9506 (compressive strength vs. bulk density). This confirms that higher bulk density and UPV values contribute to improved strength development, reinforcing PSA’s potential as a sustainable cement replacement. This result aligns with previous related studies [23,24,65,67].

### 3.3. Effects of PSA on the Durability Properties of Mortar

#### 3.3.1. Water Absorption and Porosity/Voids

**Figure 14:**
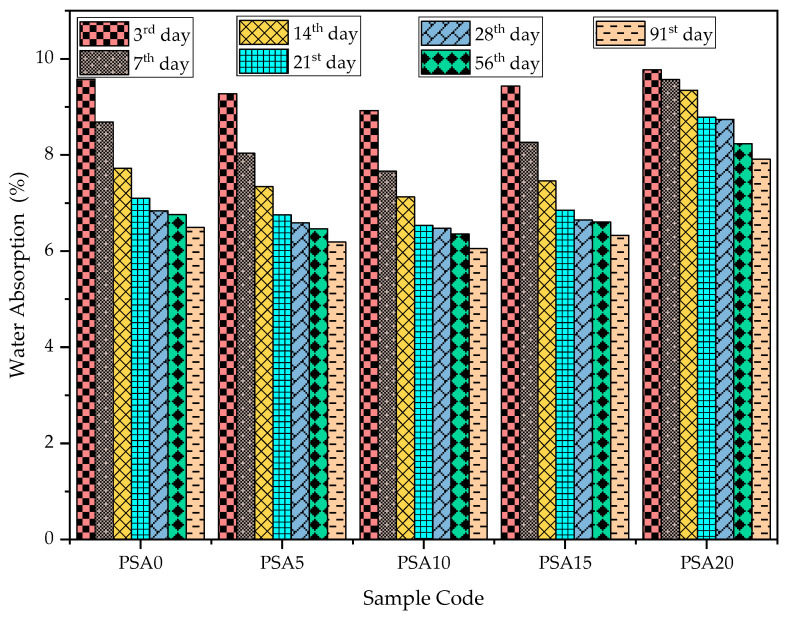
Water absorption results of PSA-incorporated mortars.

The water absorption results with different PSA replacements at various curing ages are shown in Figure 14. The data indicate that water absorption decreased with increasing PSA content up to 15% cement replacement, after which absorption levels began to rise. Compared to the control sample, 5%, 10%, and 15% PSA replacements resulted in reductions of 7.45%, 11.74%, and 4.83% at 7 days; 3.61%, 5.31%, and 2.78% at 28 days; 4.36%, 5.93%, and 2.26% at 56 days; and 4.68%, 6.80%, and 2.52% at 91 days, respectively. Conversely, the 20% PSA mix exhibited an increase in water absorption, with values rising by 10.21%, 27.81%, 21.79%, and 21.84% at 7, 28, 56, and 91 days, respectively, compared to the control mix.

The reduction in water absorption can be attributed to the continued pozzolanic activity and further hydration of cement, leading to pore refinement. PSA’s fine particle size and calcium carbonate content contribute to matrix densification, effectively reducing porosity. With extended curing, hydration processes produce additional C-S-H gels, which fill the pores and enhance the mortar’s resistance to water absorption [24,37]. The high siliceous content of PSA further promotes secondary C-S-H gel formation through reactions with calcium hydroxide, enhancing the mortar’s compactness and durability.

Related studies have reported that paint sludge powder can be used in the production of cement-based materials, confirming that water absorption can be optimized [19,44]. Beyond the optimum limit, a higher paint sludge powder content increases water absorption, suggesting that excessive replacement disrupts the matrix structure. The formation of a membrane layer by paint sludge powder may contribute to improved resistance against water and chemical ingress, despite its inherently lower porosity compared to other cementitious materials. 

Notably, the incinerated PSA used in this study yielded better water absorption resistance than uncalcined forms of paint sludge powder, as reported in studies involving lightweight mortars and ceramic production [19,44]. Furthermore, heat treatment (e.g., sintering) of uncalcined sludge powder has also been found to enhance absorption performance in ceramic applications [44]. These comparisons underscore the superior performance of incinerated PSA compared to raw paint sludge waste in terms of reducing water absorption.

**Figure 15:**
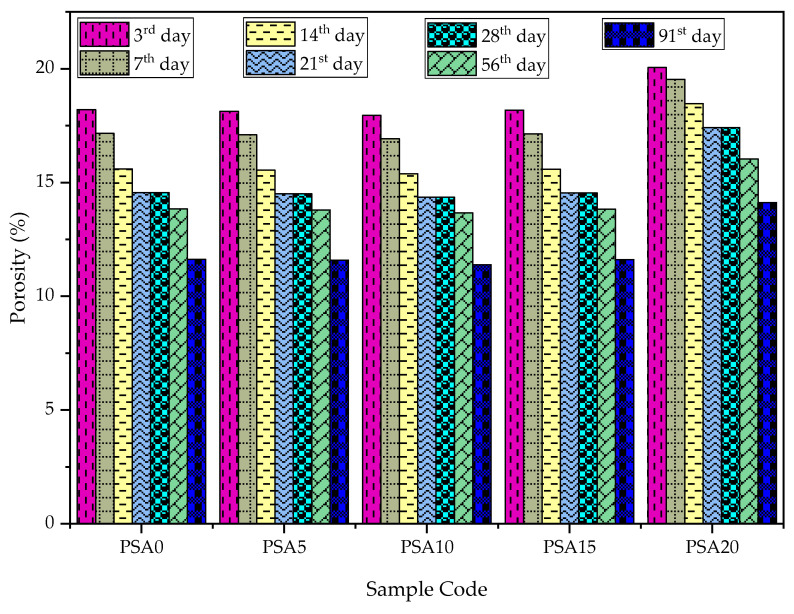
Porosity results of PSA-containing mortars.

The porosity of PSA-incorporated mortars at different curing times is illustrated in Figure 15. Similar to water absorption, porosity decreased with PSA replacement up to 15% and then increased at higher replacement levels. The 20% PSA mix exhibited 69.02% and 74.38% higher porosity than the control mix at 28 and 91 days, respectively, reinforcing the trend of increasing porosity beyond the optimal replacement level.

Previous studies have confirmed that excess waste latex paint increases porosity, leading to a decrease in both strength and durability performance due to the formed pores [33]. The observed improvement in pore structure at optimal PSA levels is primarily due to the formation of additional C-S-H gel, which enhances packing density and reduces voids. As fineness increases, unreacted particles become more uniformly distributed, further minimizing pores and strengthening the matrix [24,37,71]. The filler effect of waste latex paint enhances particle packing efficiency, thereby lowering total porosity and improving the material’s long-term durability [33].

**Figure 16:**
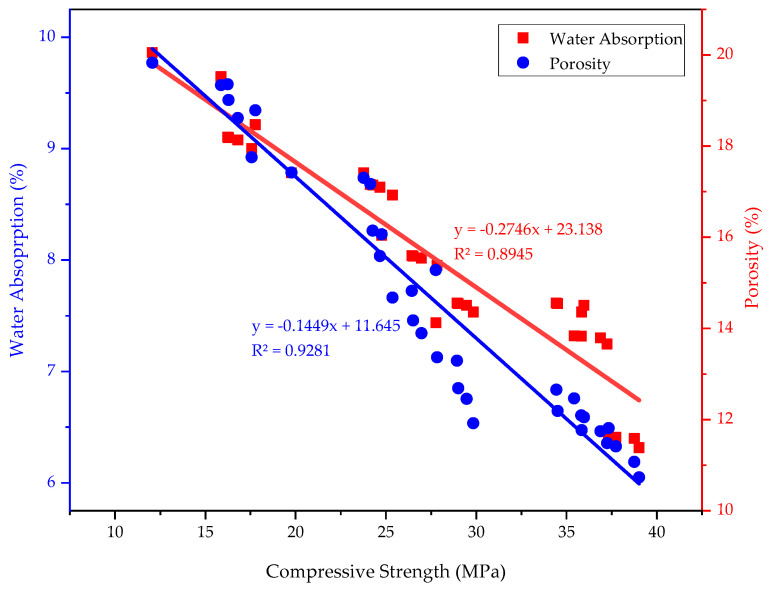
Correlation among compressive strength, water absorption, and porosity of PSA-containing mortars.

Overall, the durability performance results demonstrate the association between compressive strength, water absorption, and porosity, as shown in Figure 16. The data indicate an inverse correlation, meaning that as water absorption and porosity increase, compressive strength decreases. This inverse relationship suggests that higher water absorption results in increased porosity, which reduces the density and compactness of the mortar, ultimately leading to lower strength. The presence of more voids and interconnected pores weakens the structural integrity of the mortar, affecting both its mechanical and durability performance.

Similar correlations have been observed in other cement replacement materials in previous studies, demonstrating that higher porosity and water absorption levels correspond to reduced compressive strength and UPV values [24,65]. These findings highlight the critical role of microstructural densification in improving mortar performance and confirm that optimal replacement enhances durability by reducing porosity and improving pore refinement.

#### 3.3.2. Sulfate Attack Resistance

**Figure 17:**
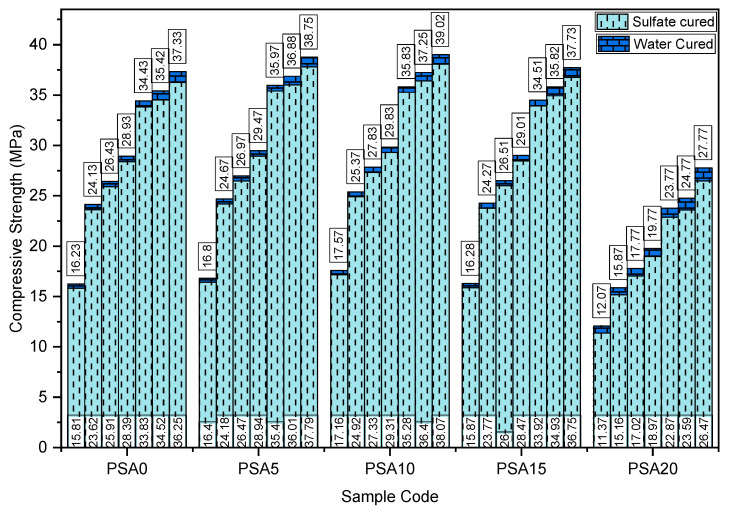
Compressive strength loss due to sulfate attack of PSA-incorporated mortars.

The sulfate resistance of mortars was evaluated by measuring the loss in compressive strength between specimens cured in pure water and those exposed to sulfate solutions. As illustrated in Figure 17, the compressive strength of mortars subjected to sulfate environments was consistently lower than that of their water-cured counterparts across all curing ages. However, the extent of this strength loss varied significantly with the level of PSA incorporation.

Mortars containing 5%, 10%, and 15% PSA replacement exhibited strong resistance to sulfate attack, outperforming the control sample at all curing ages. However, at the 15% PSA replacement, sulfate resistance showed a slight decline compared to the 5% and 10% replacements. This suggests that while PSA can enhance sulfate resistance up to an optimal level, exceeding 15% PSA in the mix may compromise the mortar’s durability in sulfate-rich environments. 

The superior sulfate resistance observed in the 5%, 10%, and 15% PSA mortars can be attributed to the pozzolanic reactions occurring between the siliceous content of PSA and the calcium hydroxide released during cement hydration. This reaction leads to the formation of additional C-S-H gel, which enhances the morphology and density of the mortar, thereby improving its resistance to sulfate ion ingress. The reduced permeability of these mortars further limits the diffusion of sulfate ions, mitigating the expansion and cracking typically associated with sulfate attack.

However, at a 20% replacement of PSA, the mortar’s increased porosity and water absorption result in higher permeability, making it more vulnerable to sulfate-induced damage. The findings underscore that while PSA can be an effective partial replacement for cement, careful optimization of the replacement percentage is essential to maintain long-term durability.

These results are consistent with those of a previous study [32] that reported the beneficial role of treated paint sludge in enhancing mortar performance in sulfate-rich environments. However, the results also underscore the importance of optimizing the replacement level. This implies that when PSA content exceeds 15%, the detrimental effects on sulfate resistance outweigh the benefits. Therefore, for applications involving sulfate exposure, PSA replacement levels should be limited to 15% or less to ensure long-term durability.

### 3.4. Effects of PSA on the Microstructural Properties of Mortar

#### 3.4.1. Scanning Electron Microscopy (SEM)

SEM analysis was carried out to examine the morphology, particle size, and microstructural development of PSA-incorporated mortar at different curing ages. 

**Figure 18:**
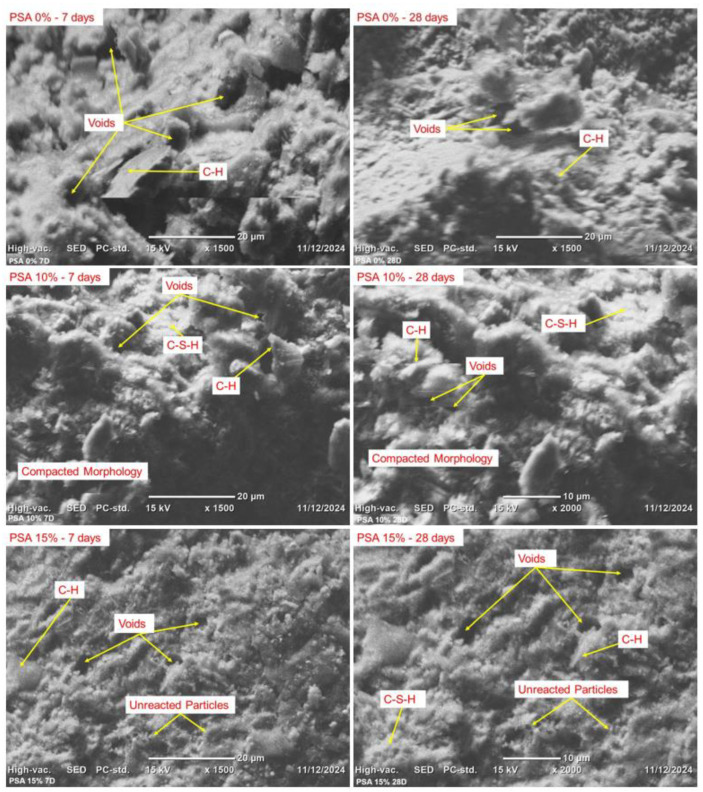
SEM images of 0%, 10%, and 15% PSA mortar at 7 and 28 days of curing.

Figure 18 depicts the SEM images of 0%, 10%, and 15% PSA mortar at 7 and 28 days of curing. As shown in Figure 17, the 10% PSA mix exhibited a denser and more compact microstructure, with C-S-H gel formation and fewer visible voids. In contrast, the 15% PSA mortar sample displayed more unreacted particles and a higher presence of voids, indicating a slightly reduced efficiency in hydration reactions compared to the 10% PSA mix. Furthermore, the control sample exhibited an even higher volume of voids, reinforcing the positive influence of PSA incorporation on matrix densification. 

The enhanced microstructural characteristics observed in 10% PSA mortar can be attributed to two primary factors. First, the higher specific surface area of PSA promotes greater pozzolanic activity, facilitating secondary C-S-H gel formation, which contributes to a more refined pore structure. Second, the filler effect of PSA allows finer particles to occupy void spaces, effectively reducing porosity and enhancing overall matrix density. The chemical composition analysis of PSA further supports these findings, demonstrating a notable siliceous content that plays a critical role in improving hydration product formation [24,37].

#### 3.4.2. X-Ray Diffraction (XRD)

**Figure 19:**
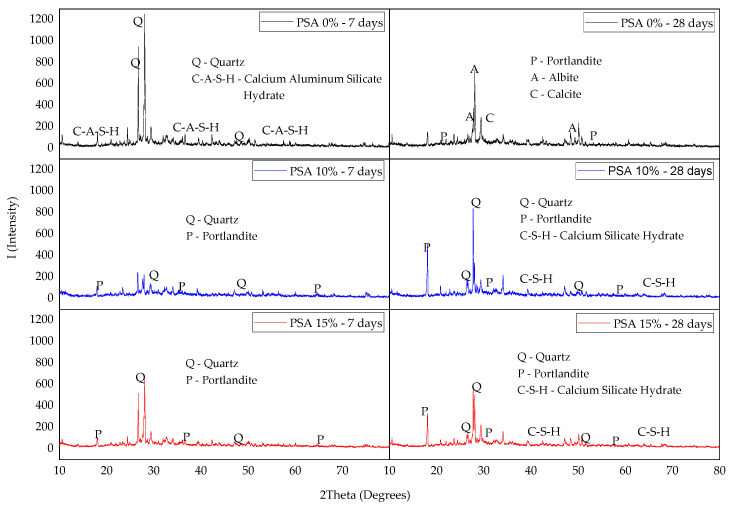
XRD pattern of 0%, 10%, and 15% PSA mortar at 7 and 28 days of curing.

An XRD analysis was performed to investigate the mineralogical composition of PSA-incorporated mortar at 7 and 28 curing days, utilizing the random powder method at 2θ of 10°–80°. Figure 19 presents the XRD patterns for 0%, 10%, and 15% PSA mortar samples.

As shown in Figure 19, the 10% PSA and 15% PSA mixes primarily consisted of quartz (SiO_2_), portlandite (C-H), and calcium silicate hydrate (C-S-H). Quartz appeared in a crystalline structure, while portlandite and C-S-H were identified in an amorphous form. The presence of C-S-H gel indicates active pozzolanic reactions, which contribute to the matrix densification and strength enhancement of PSA-incorporated mortar.

The control sample (0% PSA) exhibited quartz (SiO_2_), calcium aluminum silicate hydrate (C-A-S-H), portlandite (C-H), albite (NaAlSi_3_O_8_), and calcite (CaCO_3_), existing in both amorphous and crystalline phases. The presence of C-A-S-H in the control sample suggests a different hydration mechanism compared to PSA-containing mixes, in which the incorporation of PSA enhances pozzolanic reactions and promotes additional C-S-H formation.

These findings suggest that the incorporation of up to 10% PSA enhances the formation of hydration products, contributing to a stronger and denser matrix. However, at 15% replacement, although C-S-H formation is still evident, the presence of unreacted phases indicates reduced pozzolanic efficiency, which aligns with the observed microstructural results.

#### 3.4.3. Thermogravimetric Analysis (TGA) and Differential Thermal Analysis (DTA)

**Figure 20:**
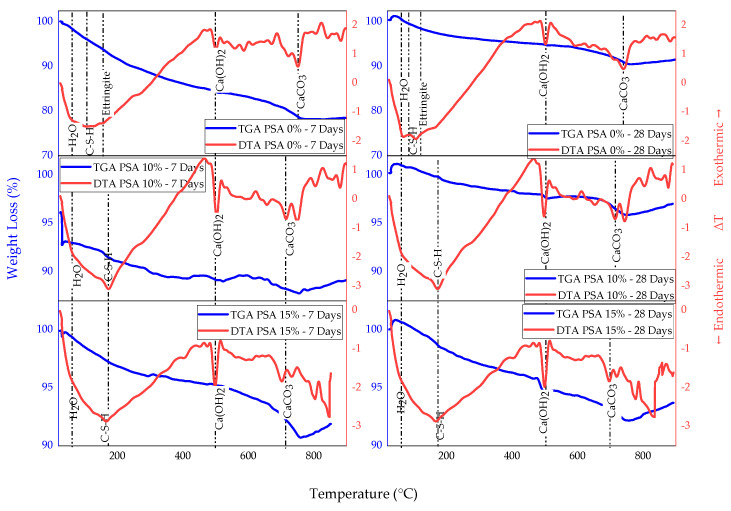
TGA/DTA results of PSA-incorporated mortars.

TGA and DTA were conducted to evaluate the thermal stability and decomposition behavior of PSA-incorporated mortar. The TGA curve, as presented in Figure 20, demonstrates the weight loss of mortar samples at different temperature ranges, corresponding to specific thermal reactions. The first weight loss occurs between 25 °C and 100 °C, which is attributed to the evaporation of absorbed water, interlayer water, and capillary moisture. Between 100 °C and 450 °C, the dehydration of C-S-H gel and ettringite occurs. Portlandite (C-H) decomposes between 480 °C and 520 °C, while the final stage of weight loss is observed between 740 °C and 760 °C, corresponding to the decarbonization of calcite (CaCO_3_). 

The total weight loss of mortar samples at 7 and 28 days is summarized in Table 7. At both curing ages, PSA-blended mortars exhibited significantly lower mass loss compared to the control. For instance, at 28 days, the 10% and 15% PSA mortars showed mass losses of 4.13% and 7.82%, respectively, compared to 9.5% for the control. These results demonstrate that PSA enhances the thermal stability of mortar, likely due to its contribution to the formation of more thermally stable hydration products and a denser microstructure.

The DTA results in Figure 20 further validate these observations. The first peak at 70 °C corresponds to the removal of free and chemically bound water molecules, while a secondary peak at 145 °C suggests the presence of ettringite. A notable C-S-H decomposition peak is observed at 170 °C, with 10% and 15% PSA exhibiting stronger signals than 0% PSA, indicating enhanced pozzolanic activity in the PSA-containing mortars. Additionally, the early dehydration of C-S-H and ettringite is more pronounced in the 0% PSA mix, whereas 10% and 15% PSA mortars exhibit a more gradual decomposition. 

The decomposition of portlandite (Ca(OH)_2)_ was detected at 495 °C across all samples, confirming consistent calcium hydroxide content. Finally, calcite decomposition occurred earlier at 700 °C and 715 °C in the 10% and 15% PSA mixes, respectively, whereas in the control sample, it was delayed until 745 °C. The earlier decomposition in PSA-modified samples suggests altered carbonation reactions due to PSA incorporation, further supporting its influence on mortar microstructure and durability. A summary of the thermal stability and decomposition behavior of PSA-modified mortars is presented in Table 8.

#### 3.4.4. Fourier-Transform Infrared Spectroscopy (FTIR)

**Figure 21:**
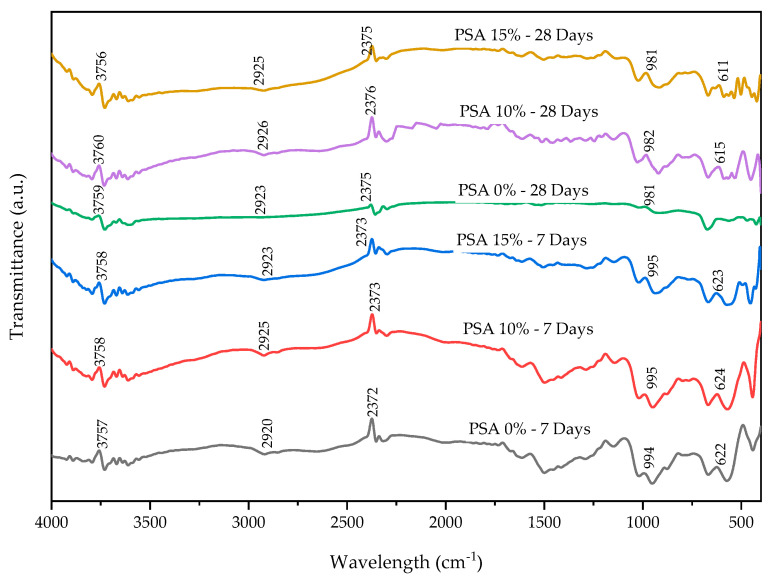
FTIR results of PSA-incorporated mortars.

FTIR was performed to analyze the chemical bonding and hydration characteristics of PSA-incorporated mortar at different curing ages. Figure 21 presents the FTIR spectra for 0%, 10%, and 15% PSA at 7 and 28 days of curing, highlighting key absorption bands that indicate the presence of hydration products. 

The dominant absorption bands were observed in the ranges of 611–624 cm^−1^, 981–995 cm^−1^, 2372–2376 cm^−1^, 2920–2926 cm^−1^, and 3756–3760 cm^−1^ across all curing periods. The presence of C-S-H is identified in the range of 485 to 1100 cm^−1^, attributed to the vibrations of Si-O bonds in the C-S-H phase [24,65]. The wave numbers in the spectra of 10% and 15% PSA samples indicate the formation of a significant amount of high-density C-S-H gels. At 7 days of curing, the C-H bond in CH_2_ or CH_3_ form appeared at 2372 cm^−1^ in 0% PSA samples and at 2373 cm^−1^ in 10% and 15% PSA samples, while at 28 days of curing, the wavenumber slightly shifted to 2375 and 2376 cm^−1^, indicating continued hydration activity. The H_2_O stretching of O-H-O was detected between 2920 and 2925 cm^−1^ at 7 days, shifting slightly to 2923–2926 cm^−1^ at 28 days, with a decrease in intensity as PSA content increased. This reduction in intensity suggests that the hydration of SiO_2_ with C-H leads to continued pozzolanic activity.

The FTIR spectra confirm that PSA incorporation enhances the hydration process by forming additional C-S-H gel, which leads to a more refined and denser microstructure. The reduced band intensity of the 15% PSA samples at 3756 cm^−1^ after 28 days suggests a lower presence of free hydroxyl groups, indicating improved hydration and matrix densification. The findings from FTIR, TGA/DTA, and XRD collectively highlight the beneficial effects of PSA on chemical hydration, thermal stability, and microstructural refinement in mortar production.

## 4. Conclusions

This study aimed to develop ecofriendly mortar by utilizing industrial waste paint sludge ash as a partial cement replacement. The conclusions drawn from the findings on material characterization, fresh properties, mechanical performance, durability, and microstructural analysis are as follows:

- Material Characterization: PSA powder was categorized as Class N pozzolan material according to ASTM C618. Its angular, irregular, and porous surface influences its behavior in cementitious mixtures.
- Fresh Properties: As PSA content increased, workability decreased, and setting time was shortened due to its higher fineness, irregular rough texture, and porous structure.
- Mechanical Properties: PSA incorporation enhanced compressive strength, UPV, and bulk density up to a 15% replacement of PSA. However, the 15% PSA replacement exhibited a slight decline compared to the 5% and 10% PSA replacements, with the 10% PSA replacement achieving the highest strength and densification.
- Durability Performance: Mortars with up to 15% PSA replacement showed improved water absorption, sulfate resistance, and lower porosity, primarily due to the pore-filling effect of the finer PSA particles.
- Microstructural Analysis: Mortars with 10% PSA exhibited higher matrix density, greater thermal stability, and minimal mass loss at elevated temperatures, indicating enhanced hydration product formation and structural integrity.
- Sustainability Impact: The effective use of PSA in mortar production helps mitigate environmental issues associated with paint sludge disposal and contributes to reducing the carbon footprint of cement manufacturing. Additionally, replacing cement with PSA can lower production costs and eliminate expenses related to sludge management, including transportation, landfill use, and land acquisition.

In summary, the findings confirm that PSA can serve as a viable cement substitute in ecofriendly mortar formulations, offering significant technical and environmental advantages without compromising performance or durability.

## Figures and Tables

**Table 1:** Oxide composition of OPC and PSA.

Oxides	OPC	PSA-700 °C	PSA-750 °C	PSA-800 °C
SiO_2_	21.74	19.73	17.65	16.48
Al_2_O_3_	4.98	21.69	25.19	19.85
Fe_2_O_3_	3.99	26.84	29.49	30.13
CaO	64.5	2.92	5.98	7.09
MgO	1.67	1.52	1.77	1.81
Na_2_O	0.19	0.12	0.12	0.12
K_2_O	0.52	0.54	0.53	0.56
Cr_2_O_3_	-	3.76	1.97	3.98
TiO_2_	-	6.39	3.20	5.5
MnO	-	1.3	1.03	1.1
P_2_O_5_	-	3.08	1.15	2.17
LOI	0.6	5.04	5.28	6.47
Others	1.81	7.07	6.64	4.74

**Table 2:** Physical properties of OPC and PSA sample.

Physical Properties	OPC	PSA
Bulk density (kg/m^3^)	1440	2157
Specific gravity (g/cm^3^)	3.15	2.76
BET surface area m^2^/g	340	490
Color	Light gray	Darker light gray

**Table 3:** Physical properties of sand.

Physical Properties	Results	Test Methods and Standards	ASTM Limit	Compliance
Unit weight (kg/m^3^)	1659.33	ASTM C29 [50]	1200–1760	✓
Relative density	2.76	ASTM C128 [51]	2.3–2.9	✓
Fineness modulus	2.7	ASTM C136 [49]	2.3–3.2	✓
Moisture content (%)	1.48	ASTM C566 [52]	0–10	✓
Absorption capacity (%)	2	ASTM C128 [51]	0.2–2	✓
Silt content (%)	2.14	ASTM C40 [53]	<5	✓

**Table 4:** Material mix proportions.

Mix Code (%)	OPC (g)	PSA (g)	Sand (g)	Water (mL)
PSA0	104.5	0.0	234.1	50.1
PSA5	99.2	5.2	234.1	50.1
PSA10	94.0	10.4	234.1	50.1
PSA15	88.8	15.7	234.1	50.1
PSA20	83.6	20.9	234.1	50.1

**Table 5:** Test methods and standards used.

Test Categories	Test Properties	Test Standards	Examined Samples	CURING Ages
Fresh properties	Slump flow	ASTM C1437 [56]	All mortar mix	_
	Consistency	ASTM C187 [57]		
	Setting time	ASTM C191 [58]		
	Soundness	ASTM C151 [59]		
Mechanical properties	Compressive strength	ASTM C109 [60]	All mortar cube specimens	3, 7, 14, 21, 28, 56, and 91 days
	UPV	ASTM C597 [61]		
	Bulk density	ASTM C138 [62]		
Durability properties	Water absorption	ASTM C642 [63]	All mortar cube specimens	3, 7, 14, 21, 28, 56, and 91 days
	Porosity	ASTM C642 [63]		
	Sulfate attack resistance	ASTM C1012 [64]		
Microstructural analysis	SEM	_	Samples taken from the mortar cube test specimens	7 and 28 days
	XRD			
	TGA/DTA			
	FTIR			

**Table 6:** SAI of PSA-incorporated mortars.

Sample Code	3rd Day	7th Day	14th Day	21st Day	28th Day	56th Day	91st Day
PSA5	1.03	1.02	1.02	1.02	1.04	1.04	1.04
PSA10	1.08	1.05	1.05	1.03	1.04	1.05	1.05
PSA15	1.00	1.01	1.00	1.00	1.00	1.01	1.01
PSA20	0.74	0.66	0.67	0.68	0.69	0.70	0.74

**Table 7:** Total weight loss of mortar samples at 7 and 28 days.

Sample Code	Weight Loss			
	7 Days		28 Days	
	(mg)	(%)	(mg)	(%)
PSA0	0.22	21.75	0.10	9.5
PSA10	0.08	8.32	0.04	4.13
PSA15	0.09	9.19	0.08	7.82

**Table 8:** Summary of the thermal stability and decomposition behavior of PSA-incorporated mortar.

Thermal Event	Temperature Range
Evaporation of absorbed water, interlayer water, and capillary moisture	25–100 °C
Dehydration of C-S-H gel and ettringite	100–450 °C
Decomposition of portlandite Ca(OH)_2_	480–520 °C
Decarbonization of calcite (CaCO_3_)	740–760 °C

## Data Availability

The data supporting the findings of this study are available upon request.
